# INSTITUTIONAL EXPERIENCES OF RACIAL DISCRIMINATION AND COGNITIVE FUNCTION: INSTRUMENTAL VARIABLE ANALYSIS

**DOI:** 10.1093/geroni/igad104.2008

**Published:** 2023-12-21

**Authors:** Jourdyn Lawrence, Ichiro Kawachi

**Affiliations:** Dornsife School of Public Health, Drexel University, Philadelphia, Pennsylvania, United States; Harvard University, Boston, Massachusetts, United States

## Abstract

In addition to enduring the ongoing and persistent legacy of structural racism in the United States, Black adults often encounter sources of social stress, like racial discrimination, that have documented associations with health outcomes. As inequities in cognitive aging persist for Black adults, research has worked to examine modifiable factors, like racial discrimination, that may serve as the basis for intervention development. The dysregulation of inflammatory biomarkers has been proposed as one pathway through which racial discrimination becomes embodied to contribute to racial inequities in cognitive function. We assessed how racial discrimination occurring in institutional settings affects cognitive function among a sample of Black and white adults from the Coronary Artery Risk Development in Young Adults (CARDIA) study. To mitigate the influences of measurement error and unmeasured or residual confounding on our findings, we used an instrumental variable analysis to estimate the unbiased, causal association between racial discrimination in institutional settings and a battery of standardized cognitive function measures. We found that increased experiences of racial discrimination had adverse impacts on cognitive function. Understanding how racial discrimination in these settings influences cognitive outcomes supplies insight into points of potential intervention at institutional and policy levels.

